# Anti-cancer effects of newly developed chemotherapeutic agent, glycoconjugated palladium (II) complex, against cisplatin-resistant gastric cancer cells

**DOI:** 10.1186/1471-2407-13-237

**Published:** 2013-05-14

**Authors:** Mamoru Tanaka, Hiromi Kataoka, Shigenobu Yano, Hiromi Ohi, Keisuke Kawamoto, Takashi Shibahara, Tsutomu Mizoshita, Yoshinori Mori, Satoshi Tanida, Takeshi Kamiya, Takashi Joh

**Affiliations:** 1 Departments of Gastroenterology and Metabolism, Nagoya City University Graduate School of Medical Sciences, Kawasumi, Mizuho-cho, Mizuho-ku, Nagoya, 467-8601, Japan; 2 Graduate School of Materials Science, Nara Institute of Science and Technology, 8916-5 Takayama, Ikoma, Nara, 630-0192, Japan; 3 Office of Society-Academia Collaboration for Innovation, Kyoto University, Katsura, Nishikyo-ku, Kyoto, Japan; 4 Department of Industrial Chemistry, Kinki Polytechnic College, 1778 Inaba-cho, Kishiwada, Osaka, 596-0103, Japan; 5 Department of Chemistry, Okayama University of Science, 1-1Ridai-cho, Kita-ku, Okayama, 700-0005, Japan

**Keywords:** Glycoconjugated platinum (II) complex, Glycoconjugated palladium (II) complex, Cisplatin, Drug resistance, Gastric cancer

## Abstract

**Background:**

Cisplatin (CDDP) is the most frequently used chemotherapeutic agent for various types of advanced cancer, including gastric cancer. However, almost all cancer cells acquire resistance against CDDP, and this phenomenon adversely affects prognosis. Thus, new chemotherapeutic agents that can overcome the CDDP-resistant cancer cells will improve the survival of advanced cancer patients.

**Methods:**

We synthesized new glycoconjugated platinum (II) and palladium (II) complexes, [PtCl_2_ (L)] and [PdCl_2_ (L)]. CDDP-resistant gastric cancer cell lines were established by continuous exposure to CDDP, and gene expression in the CDDP-resistant gastric cancer cells was analyzed. The cytotoxicity and apoptosis induced by [PtCl_2_ (L)] and [PdCl_2_ (L)] in CDDP-sensitive and CDDP-resistant gastric cancer cells were evaluated. DNA double-strand breaks by drugs were assessed by evaluating phosphorylated histone H2AX. Xenograft tumor mouse models were established and antitumor effects were also examined *in vivo*.

**Results:**

CDDP-resistant gastric cancer cells exhibit ABCB1 and CDKN2A gene up-regulation, as compared with CDDP-sensitive gastric cancer cells. In the analyses of CDDP-resistant gastric cancer cells, [PdCl_2_ (L)] overcame cross-resistance to CDDP *in vitro* and *in vivo*. [PdCl_2_ (L)] induced DNA double-strand breaks.

**Conclusion:**

These results indicate that [PdCl_2_ (L)] is a potent chemotherapeutic agent for CDDP-resistant gastric cancer and may have clinical applications.

## Background

Cancer is a leading cause of death worldwide, and according to the WHO mortality database (as at November 2006), gastric cancer is the second leading cause of cancer death after lung cancer.

Cisplatin (CDDP) is the most frequently used chemotherapeutic agent for various types of advanced cancer and is used in combination regimens. Some CDDP-based combination chemotherapy regimens have also shown high response rates [1]. Based on recent Japanese phase III trials for metastatic gastric cancer, S1 plus cisplatin combination chemotherapy was established as the standard first-line chemotherapy [2].

However, CDDP-based combination chemotherapy regimens have several disadvantages, including side effects such as nephrotoxicity, neurotoxicity, ototoxicity and vomiting. In addition, some tumors acquire resistance to CDDP, reducing its efficacy [3,4]. Several mechanisms are involved in CDDP resistance [5]. Such mechanisms include decreased intracellular drug accumulation and/or increased drug efflux [6-9], drug inactivation by increased levels of cellular thiols [6,10], increased nucleotide excision-repair activity [9,11] and evasion of apoptosis [6,12]. Thus, for continued progress in cancer therapy, more effective drugs must be found.

Cancer cells take in higher levels of glucose than normal cells, a phenomenon known as the Warburg effect [13]. To achieve lower undesired toxicity, enhanced solubility and tumor selectivity, we have developed and have reported several glycoconjugated drugs [14,15]. Another strategy to design new antitumor agents related to CDDP is to change the nature of the central metal ion [16,17]. As palladium (Pd) chemistry is similar to that of platinum (Pt), Pd complexes (II) are expected to exhibit antitumor activities similar to those of Pt. Attempts have been made to synthesize Pd (II) complexes with such activities, as Pd complexes are expected to have less kidney toxicity than Pt complexes [18].

In this study, we synthesized a new glycoconjugated Pt (II) complex and a new glycoconjugated Pd (II) complex, and analyzed its cytotoxicity, ability to induce apoptosis, and ability to induce DNA double-strand breaks in CDDP-sensitive and CDDP-resistant gastric cancer cell lines *in vitro* and *in vivo*.

## Methods

### Drugs

Reagents and solvents used in this study were commercial products of the highest available purity. The Pt (II) and Pd (II) complexes were easily prepared using the one-pot reaction of Pt (II) or Pd (II) salt, amino sugar and pyridine aldehyde derivative without isolation of a Schiff base ligand (L) as follows.

[PtCl_2_ (L)] (L = 2-deoxy-2-[(2-pyridinylmethylene)amino]-α-D-glucopyranose):Dichloro (2-deoxy-2-[(2-pyridinylmethylene)amino]-α-D-glucopyranose) Pt. An aqueous (50 mL) solution of D (+)-glucosamine • hydrochloride (0.65 g, 3.0 mmol) was neutralized with NaHCO_3_ (0.26 g, 3.1 mmol). To this solution, a MeOH (50 mL) solution of 2-pyridinecarbaldehyde (0.32 g, 3.1 mmol) was added, followed by stirring for 2 h and addition of K_2_ [PtCl_4_] (1.25 g, 3.0 mmol) in 30 mL of H_2_O. The reaction was continued for another 41 h at room temperature. The mixture was concentrated by evaporation and the resulting residue was purified by silica gel column chromatography (eluent: acetone) to give a pale yellow powder (1.07 g, 67%). Single crystals were obtained by recrystallization from MeOH/Et_2_O. Anal. calcd for [PtCl_2_ (L)], C_12_H_18_Cl_2_N_2_O_5_Pt, C; 26.98, H; 3.02, N; 5.24. found for C; 27.13, H; 2.97, N; 5.07. MS (FAB, pos): *m*/*z* = 498 [M–Cl]^+^.

[PdCl_2_ (L)] (L = 2-deoxy-2-[(2-pyridinylmethylene)amino]-α-D-glucopyranose):Dichloro (2-deoxy-2-[(2-pyridinylmethylene)amino]-α-D-glucopyranose) palladium. This complex was prepared by following a similar procedure as described above for [PtCl_2_ (L)] using Na_2_ [PdCl_4_] instead of K_2_ [PtCl_4_]. The complex was dissolved in MeOH and insoluble materials were removed by filtration. The filtrate was concentrated by evaporation to give a pale yellow powder (1.1 g, 83%). This complex was purified by recrystallization from MeOH/Et_2_O. Anal. calcd for [PtCl_2_ (L)], C_12_H_18_Cl_2_N_2_O_5_Pd, C; 32.35, H; 3.62, N; 6.29. found for C; 32.02, H; 3.51, N; 6.01. MS (FAB, pos): *m*/*z* = 431 [M–HCl + Na]^+^.

CDDP and CBDCA were purchased from Bristol-Myers Co. (Tokyo, Japan). L-OHP was purchased from Yakult (Tokyo, Japan).

### Measurements

Elemental analysis was carried out on a Perkin-Elmer 240C or a Fisons Instruments EA1108 Elemental Analyzer. ^1^H- and ^13^C-NMR spectra were recorded on a JEOL JNM-GSX400 in *N*,*N*-dimethylformamide-d_7_ (DMF-d_7_)/D_2_O. Mass spectra were obtained on a JEOL JMS-700 T Tandem MS-station mass spectrometer.

### Crystallography

Suitable crystals for X-ray crystallography were obtained by slow recrystallization of [PtCl_2_ (L)] and [PdCl_2_ (L)] from a minimal amount of methanol and ether mixtures. Crystallographic data (excluding structure factors) for the structure reported in this paper were deposited with the Cambridge Crystallographic Data Center as supplementary publication no. CCDC-835397. Copies of the data can be obtained free of charge on application to CCDC, 12 Union Road, Cambridge CB21EZ, UK (Fax: (+44) 1223-336-033; E-mail: deposit@ccdc.cam.ac.uk).

### Cell culture

The human gastric cancer cell lines MKN28 (Japanese Cancer Research Resources Bank, No. 0253) and MKN45 (Japanese Cancer Research Bank, No. 0254) were cultured in RPMI1640 (Sigma-Aldrich, St. Louis, MO) supplemented with 10% fetal bovine serum (FBS) and 1% ampicillin and streptomycin. Cells were cultured under an atmosphere of 5% CO_2_ at 37°C.

### Establishment of CDDP-resistant sublines from MKN28 and MKN45

CDDP-resistant MKN28 (MKN28 (CDDP)) and CDDP-resistant MKN45 (MKN45 (CDDP)) were established by continuous exposure to CDDP starting at 0.5 μmol/L and increasing in a stepwise manner to 10 μmol/L for more than 5 months. Experiments with these sublines were performed after maintenance in CDDP-free medium for 2–3 weeks.

### RT2 Profiler PCR arrays for human cancer drug resistance & metabolism

Total RNA (1 μg) from MKN45 (0) or MKN45 (CDDP) was converted to cDNA and used to screen inflammatory cytokines and receptors using quantitative real-time PCR arrays according to the manufacturer’s instructions (SuperArray Bioscience). Reactions were cycled in an ABI Prism 7500 FAST sequence detector (Applied Biosystems) and acquired data were analyzed using the DDCt method to determine the expression levels of each transcript normalized against the expression level of housekeeping gene controls. A gene-wise, two-sample *t*-test was performed for each transcript to identify statistical differences in expression between MKN45 (0) or MKN45 (CDDP).

### *In vitro* treatment

Cell viability was determined by WST-8 cell proliferation assay. Gastric cancer cells were seeded into 96-well culture plates at 5 × 10^3^ cells/100 μL/well and incubated overnight. Cells were treated for 48 h with graded concentrations of CDDP (0–200 μmol/L), [PtCl_2_(L)] (0–200 μmol/L), [PdCl_2_(L)] (0–200 μmol/L), L-OHP (0–100 μmol/L) or CABDA (0–400 μmol/L). After treatment, cells were incubated with cell a counting kit-8 (Dojindo, Kumamoto, Japan) for 4 h and absorption at 450 nm was measured with a microscope reader (SPECTRA MAX340; Molecular Devices, Silicon Valley, CA). Cell viability was expressed as a percentage vs. untreated control cells and half maximal (50%) inhibitory concentration (IC_50_) was calculated. Resistance factor (RF) is defined as the relative ratio of IC_50_ values in both cell lines (MKN28 (CDDP)/MKN28 (0) or MKN45 (CDDP)/MKN45 (0)).

### Assessment of apoptosis

Apoptosis was assessed by analysis of activation of caspase-3 and caspase-7 using the substrate DEVD-aminoluciferin from the Caspase-Glo 3/7 Assay kit (Promega) according to the manufacturer’s instructions. Briefly, gastric cancer cells (10^4^ per well) were plated on a 96-well culture plate with three replicates per treatment. After 24 h of plating, cells were treated for 72 h with graded concentrations of CDDP (0–200 μmol/L), [PtCl_2_(L)] (0–200 μmol/L), [PdCl_2_(L)] (0–200 μmol/L), L-OHP (0–100 μmol/L) or CABDA (0–400 μmol/L). Caspase-Glo reagent was added to each well and incubated for 1 h, and luminescence was measured using a LUMAT LB 9507 luminometer (Berthold Technologies). Results were analyzed by Welch’s *t*-test between MKN45 (0) and MKN45 (CDDP).

### Assessment of DNA double-strand breaks

Cells were washed with PBS (−) and subsequently dissolved in 1 cell lysis buffer (Cell Signaling Technology) containing 20 mmol/L Tris–HCl (pH 7.5), 150 mmol/L NaCl, 1 mmol/L Na2EDTA, 1 mmol/L EGTA, 1% Triton, 2.5 mmol/L sodium pyrophosphate, 1 mmol/L h-glycerophosphate, 1 mmol/L Na3VO4, and 1 Ag/mL leupeptin with the addition of 1 mmol/L phenylmethylsulfonyl fluoride. After disruption in an ice bath using a Bio-ruptor sonicator (Cosmo Bio) for 15 s, lysates were centrifuged at 15,000 rpm for 10 min at 4°C. Each sample was normalized as equal protein concentrations using a protein assay kit (Bio-Rad Laboratories). An equal quantity of 2 SDS-PAGE sample buffer [0.5 mol/L Tris–HCl (pH 7.2), 1% SDS, 100 mmol/L β-mercaptoethanol, and 0.01% bromophenol blue] was added to each sample, followed by boiling for 5 min at 100°C. Aliquots of sample were fractioned on 8% to 15% SDS-PAGE and were then electroblotted onto nitrocellulose membrane. The membrane was blocked with 5% skimmed milk in PBS (−) for 1 h at room temperature. The membrane was incubated with primary antibodies, anti-γH2AX (Bethyl Laboratories, Inc., 1:2000), overnight at 4°C and was then washed with 0.05% Tween 20 in PBS (−) three times at 5-min intervals. The membrane was incubated with secondary antibody for 1 h at room temperature followed by three washes with 0.05% Tween 20 in PBS (−) three times at 5-min intervals. The membrane was treated with enhanced chemiluminescence detection reagents (Amersham) for 1 min at room temperature and exposed to scientific imaging films (Eastman Kodak), and proteins were visualized as bands. Filters were stripped and re-probed with monoclonal β-actin antibody (Abcam plc) as an internal control.

### Animals and tumor models

Pathogen-free female nude mice (BALB/c Slc-nu/nu) aged 4 weeks and weighing 20–25 g were obtained from Japan SLC (Kyoto, Japan). Animals were allowed to acclimatize for 2 weeks in the animal facility before any interventions were initiated. Xenograft tumor models were established by subcutaneously implanting 3 × 10^6^ gastric cancer cells (MKN45 (0), MKN45 (CDDP)) in 200 μL of PBS. Experimental procedures were approved by the Nagoya City University Center for Experimental Animal Science, and mice were raised in accordance with the guideline of the Nagoya City University Center for Animal Experiments.

### *In vivo* treatment

At 7 days after tumor inoculation, mice were given an intraperitoneal injection of CDDP, [PtCl_2_ (L)] or [PdCl_2_ (L)] at a dose of 40 μmol/kg. Tumor growth was monitored daily by measuring tumor volume with vernier calipers. Tumor volume was calculated using the following formula: (length × width × depth)/2. Each group consisted of 5 mice. Results were analyzed by multiple testing (Holm method) between groups.

### Statistical analysis

Descriptive statistics and simple analyses were carried out using the statistical package R version 2.4.1 (http://www.r-project.org/). Apoptosis induction was analyzed by Welch’s *t*-test. Antitumor effects were analyzed by the Bonferroni-Holm method. P-values of <0.05 were considered to be statistically significant.

## Results

### Crystal structure of [PtCl_2_ (L)] and [PdCl_2_ (L)]

**Figure 1:**
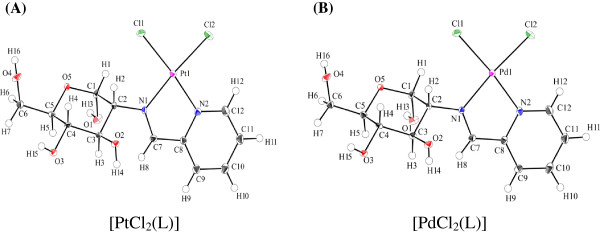
**Structures of complexes used in this study.** (**A**) Perspective drawing of [PtCl_2_ (L)] with atomic numbering scheme in the crystal. Selected bond length (Å) and angles (^o^), Pt(1)-Cl(1) 2.2985(8), Pt(1)-Cl(2) 2.2922(8), Pt(1)-N(1) 2.016(3), Pt(1)-N(2) 2.006(3); Cl(1)-Pt(1)-Cl(2) 89.77(3), Cl(1)-Pt(1)-N(1) 94.84(7), Cl(2)-Pt(1)-N(2) 95.30(7), N(1)-Pt(1)-N(2) 80.14(10). (**B**) Perspective drawing of [PdCl_2_ (L)] with atomic numbering scheme in the crystal. Selected bond length (Å) and angles (^o^), Pd(1)-Cl(1) 2.2940(8), Pd(1)-Cl(2) 2.2832(8), Pd(1)-N(1) 2.033(2), Pd(1)-N(2) 2.025(2); Cl(1)-Pd(1)-Cl(2) 91.04(3), Cl(1)-Pd(1)-N(1) 94.02(7), Cl(2)-Pd(1)- N(2) 94.50(6), N(1)-Pd(1)-N(2) 80.49(8).

The crystal structures of [PtCl_2_ (L)] and [PdCl_2_ (L)] (Cambridge Crystallographic Data Center as supplementary publication no. CCDC-835397) show that each metal atom is surrounded by four donor atoms, two nitrogen atoms and two chloride ions, in a cis-configuration. As expected, the geometry around the metal center is approximately square planar (Figure 1). The pyranoid ring of the sugar unit adopts an unusual α-^4^C_1_ conformation. Thus, both complexes have similar structures.

### Conformational analysis of sugar units on [PtCl_2_ (L)] and [PdCl_2_ (L)] by means of NMR measurements

^1^H-NMR and ^13^C-NMR spectra of the two complexes were obtained in DMF-d_7_/D_2_O and unambiguously assigned by ^1^H-^1^H and ^13^C-^1^H COSY two-dimensional NMR spectroscopy. Conformation of the sugar ring in both complexes was investigated by ^1^H-NMR spectroscopy in DMF-d_7_/D_2_O after OH proton exchange, which reveals signals originating from protons that are attached to the carbon atoms of the sugar unit. The vicinal proton-proton coupling constants for [PtCl_2_ (L)] (^3^ *J*_1,2_ = 3.2, ^3^ *J*_2,3_ = 11.2, ^3^ *J*_3,4_ = 8.4, ^3^ *J*_4,5_ = 10.0 Hz) and [PdCl_2_ (L)] (^3^ *J*_1,2_ = 3.4, ^3^ *J*_2,3_ = 11.2, ^3^ *J*_3,4_ = 8.6, ^3^ *J*_4,5_ = 10.0 Hz) correspond to α-^4^*C*_1_ conformations as observed in the X-ray crystallography, indicating the structural similarity in the sugar unit in the solid and solution states.

### Genes up-regulated in CDDP-resistant gastric cancer sublines

The 20-fold changes in gene expression for MKN45 (0) and MKN45 (CDDP) are presented in Table 1. Among 84 genes related to human cancer drug resistance and metabolism, 8 genes were significantly altered with fold changes larger than 20. Genes that were up-regulated by greater than 20-fold were ABCB1, APC, ATM, BRCA2 and CDKN2A, whereas down-regulated genes were CYP2B6, CYP2C19 and PPARγ.

**Table 1:** Expression profiles of genes related to human cancer drug resistance and metabolism showing at least 20-fold change in expression

**Symbol**	**GenebankID**	**Incease**	**Gene name**
ABCB1	NM_000927	122.73	ABC20,CD243,CLCS,GP170,MDR1,MGC163296,P-GP,PGY1
APC	NM_000038	27.25	BTPS2,DP2,DP2.5,DP3,GS
ATM	NM_000051	27.35	AT1,ATA,ATC,ATD,ATDC,ATE,DKFZp781A0353,MGC74674,TEL1,TELO1
BRCA2	NM_000059	34.61	BRCC2,BROVCA2,FACD,FAD,FAD1,FANCB,FANCD,FANCD1
CDKN2A	NM_000077	2689.53	ARF,CDK4I,CDKN2,CMM2,INK4,INK4a,MLM,MTS1,TP16,p14,Prop14ARF,p16,p16INK4,p16INK4a,p19
CYP2B6	NM_000767	−39.27	CPB6,CYP2B,CYPIIB6,IIB1,P450
CYP2C19	NM_000769	−145.20	CPCJ,CYP2C,P450C2C,P450IIC19
PPARG	NM_015869	−29.31	CIMT1,NR1C3,PPARG1,PPARG2,PPARgamma

### [PdCl_2_ (L)] revealed minimum resistance to CDDP-resistant gastric cancer cells

We investigated the cytotoxicity of CDDP, [PtCl_2_ (L)], [PdCl_2_ (L)], L-OHP and CABDA in the gastric cancer cell lines MKN28 (0), MKN28 (CDDP), MKN45 (0) and MKN45 (CDDP), and summarize the results in Table 2. In the parent cell lines (MKN28 (0) and MKN45 (0)), [PtCl_2_ (L)] and [PdCl_2_ (L)] exhibited lower cytotoxicity than CDDP and L-OHP, and higher cytotoxicity than CABDA. Resistance factor (RF) was calculated as the relative ratio of IC_50_ values in both cell lines (MKN28 (CDDP)/MKN28 (0) or MKN45 (CDDP)/MKN45 (0)). Similarly to CABDA, cells treated with [PtCl_2_ (L)] showed cross-resistance to CDDP. On the other hand, [PdCl_2_ (L)] overcame cross-resistance to CDDP, similarly to L-OHP, although [PdCl_2_ (L)] showed a lower degree of cross-resistance than L-OHP (Table 2).

**Table 2:** ***In vitro *cytotoxicity assay in CDDP-sensitive and -resistant gastric cancer cell lines**

**MKN28**	**Resistance factor**	**IC50(μM)**	
		**MKN28(0)**	**MKN28(CDDP)**
[PdCl2(L)]	1.02	78.9 ± 4.0	80.8 ± 6.6
L-OHP	1.19	46.4 ± 4.0	55.2 ± 3.8
[PtCl2(L)]	2.54	111.7 ± 27.1	283.9 ± 19.3
CDDP	3.37	19.4 ± 2.4	65.4 ± 4.6
CABDA	4.33	202.9 ± 17.2	878.3 ± 34.1
**MKN45**	**Resistance factor**	**IC50(μM)**	
		**MKN45(0)**	**MKN45(CDDP)**
[PdCl2(L)]	1.14	61.2 ± 6.8	69.7 ± 4.1
L-OHP	1.3	27.3 ± 1.1	35.6 ± 6.7
[PtCl2(L)]	2.18	129.5 ± 14.8	282.6 ± 34.5
CDDP	3.27	23.5 ± 2.2	77.0 ± 8.5
CABDA	3.42	152.8 ± 3.7	522.0 ± 27.4

### [PdCl_2_ (L)] induced apoptosis in CDDP-resistant gastric cancer cell lines

**Figure 2:**
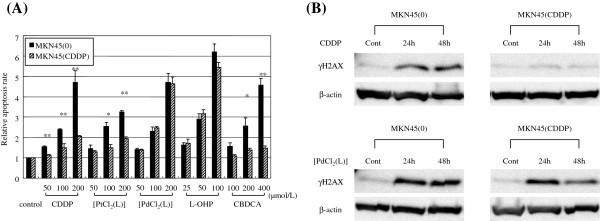
**Investigation of cytotoxicity mechanism of [PtCl_2_ (L)] and [PdCl_2_ (L)].** (**A**) [PdCl_2_ (L)] induced apoptosis on CDDP-resistant gastric cancer cell lines. Apoptosis was assessed by analyzing activation of caspase-3 and caspase-7. Mean of three independent experiments in triplicate; bars, SE. Values for apoptosis of cells in FBS alone were used as controls. Significance was determined by Welch’s *t*-test. *, P < 0.05, **, P < 0.01 relative to parental cell line. (**B**) [PdCl_2_ (L)] induced DNA double-strand breaks in CDDP-resistant gastric cancer cells. Cells were labeled with antibody against phosphorylated histone H2AX (γ-H2AX), which detects double-strand breaks caused by drugs such as CDDP. An evaluation of γ-H2AX protein expression was investigated by Western blotting at 24 or 48 h after treatment.

We examined apoptosis induction by CDDP, [PtCl_2_ (L)], [PdCl_2_ (L)], L-OHP and CABDA in the gastric cancer cell lines MKN45 (0) and MKN45 (CDDP) (Figure 2A). In the parental cell line (MKN45 (0)), all drugs tended to induce apoptosis in a dose-dependent manner. In the CDDP-resistant subline (MKN45 (CDDP)), induction of apoptosis by CDDP, CABDA and [PtCl_2_ (L)] was lower than in the parental cell line. On the other hand, [PdCl_2_ (L)] and L-OHP maintained apoptosis induction against CDDP-resistant gastric cancer cells.

### [PdCl_2_ (L)] induced DNA double-strand breaks in CDDP-resistant gastric cancer cells

Cells were labeled with an antibody against phosphorylated histone H2AX (γ-H2AX), which detects double-strand breaks caused by drugs such as CDDP [19]. We used Western blotting for evaluation of γ-H2AX protein expression by CDDP and [PdCl_2_ (L)] in the gastric cancer cell lines MKN45 (0) and MKN45 (CDDP). In the parental cell line (MKN45 (0)) treated with CDDP or [PdCl_2_ (L)], γ-H2AX protein levels increased and were the same by 24 and 48 h after treatment. In the CDDP-resistant subline (MKN45 (CDDP)), γ-H2AX protein levels increased with [PdCl_2_ (L)], but did not increase with CDDP (Figure 2B). These results indicated that [PdCl_2_ (L)], but not CDDP induced DNA double-strand breaks in CDDP-resistant gastric cancer cells.

### [PdCl_2_ (L)] significantly suppressed CDDP-resistant gastric cancer cell proliferation

**Figure 3:**
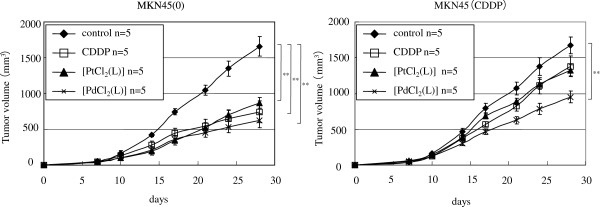
[**PdCl_2_ (L)] significantly suppressed CDDP-resistant gastric cancer cell proliferation in xenograft model.** Cells were inoculated in dorsal skin at a concentration of 3 × 10^6^ gastric cancer cells (MKN45 (0), MKN45 (CDDP)) in 200 μL of PBS. At 7 days after tumor inoculation, tumor-bearing mice were given intraperitoneal injection of CDDP, [PtCl_2_ (L)] or [PdCl_2_ (L)] at a dose of 40 μmol/kg (n = 5 for each). Tumor volumes were monitored for 28 days in control mice (no treatment), and mice treated with CDDP, [PtCl_2_ (L)] or [PdCl_2_ (L)]. Data are means ± SE. Significance was determined by the Bonferroni-Holm method. **, P < 0.01 relative to controls.

We examined the effects of CDDP, [PtCl_2_ (L)] and [PdCl_2_ (L)] on xenograft tumor models established by subcutaneously implanting the gastric cancer cell lines MKN45 (0) and MKN45 (CDDP). At 7 days after tumor inoculation, mice were given an intra-peritoneal injection of CDDP, [PtCl_2_ (L)] or [PdCl_2_ (L)] at a dose of 40 μmol/kg. In MKN45 (0) nude mice, CDDP, [PtCl_2_ (L)] and [PdCl_2_ (L)] suppressed tumor growth significantly as compared to controls (p < 0.01). In MKN45 (CDDP) nude mice, [PdCl_2_ (L)] suppressed tumor growth significantly (p < 0.01) as compared to CDDP, but [PtCl_2_ (L)] did not (Figure 3). None of the therapies had any obvious side effects, such as diarrhea or weight loss (data not shown).

## Discussion

[PtCl_2_ (L)] and [PdCl_2_ (L)] were developed as antitumor drugs with sugar conjugated ligands, and were expected to have a number of advantages, including significant reductions in side effects, improved water solubility, and greater cellular uptake. These complexes were very easily prepared in good yields by one-pot reaction of Pt or Pd salts, amino sugar and pyridine aldehyde derivative without isolation of Schiff base ligand, and were characterized by X-ray crystallography and ^1^H- and ^13^C-NMR spectra. One-pot reaction is a strategy to improve the efficiency of a chemical reaction whereby a reactant is subjected to successive chemical reactions. This saves time and resources by avoiding lengthy separation processes and purification of the intermediate chemical compounds while increasing chemical yield.

In this report, we found that gastric cancer cell lines adapted to growth in the presence of 10 μmol/L CDDP (MKN45 (CDDP)) showed enhanced ABCB1 and CDKN2A expression as compared with their CDDP-sensitive parental cell lines (MKN45 (0)) (Table 1). Prolongation of the cell cycle at the G1-S transition allows for DNA repair to occur. It is therefore unsurprising that growth arrest mediated by CDKN2A is able to enhance resistance to drugs whose mechanism of action is dependent on DNA damage, such as CDDP [20]. ABCB1 is the most extensively studied ABC transporter [21]. The expression of P-glycoprotein ABCB1 is implicated in multidrug resistance (MDR). MDR proteins confer drug resistance by reducing intracellular drug accumulation due to active efflux of drugs [22,23]. The CDDP-resistant cell line (MKN45 (CDDP)) was useful for studying the resistance mechanisms of CDDP and for studying the effects of other anticancer drugs for gastric cancer under CDDP resistance.

Many experiments have been performed in order to develop new anti-cancer drugs that show preferential accumulation within the target tumor tissue for various active targeting approaches, such as liposomes [24], polymer microspheres [25-27] and nanoparticles [28-31]. Our results indicate that the glucose-linked anticancer drug is a useful drug delivery system for accumulation in the target tumor.

In order to circumvent CDDP resistance, significant amounts of work have been devoted to preparing anticancer complexes, including amine Pt complexes [32,33], diamine Pt complexes [34,35], *trans*-Pt complexes [36-38], multinuclear Pt complexes [39-41] and Pt (IV) coordination complexes [42-44]. Progress in the field of anticancer chemistry of Pd-based transition metal complexes has been reviewed [45]. [PdCl_2_ (L)] and L-OHP overcame cross-resistance to CDDP, although [PdCl_2_ (L)] showed a lower degree of cross-resistance than L-OHP (Table 2). The cytotoxicity of L-OHP in CDDP-resistant cell lines has been considered to be due to the differences of DNA damage and/or recognition processes between CDDP and L-OHP [46]. The DNA damage caused by Pd (II) compounds is reportedly processed in a different manner from that induced by Pt (II) complexes [47]. In the CDDP-resistant subline (MKN45 (CDDP)), [PdCl_2_ (L)] showed significantly higher antitumor effects *in vitro* (Table 2) and *in vivo* (Figure 3) as compared with CDDP and [PtCl_2_ (L)]. Apoptosis by [PdCl_2_ (L)] did not decrease when compared with parental cells, although apoptosis induced by [PtCl_2_ (L)] decreased (Figure 2A). These results indicate that the resistance mechanism of Pd (II) complexes might be different from those of Pt (II) complexes.

Phosphorylation of histone H2AX (γH2AX) has been used as an indicator of exposure to a variety of DNA-damaging agents such as ionizing radiation [48], gemcitabine [49], topotecan [50], etoposide, bleomycin, and doxorubicin [51]. The stimulus for γH2AX formation after CDDP treatment is replication fork collapse and subsequent double-strand break formation at sites of inter-strand cross-links [52,53] immediately after formation of double-strand breaks [52,54]. The present results revealed that [PdCl_2_ (L)] induced DNA double-strand breaks in CDDP-resistant gastric cancer cells in which CDDP could not induce DNA double-strand breaks (Figure 2B).

## Conclusion

We demonstrated that a new glycoconjugated Pt (II) complex, [PtCl_2_ (L)], and a new glycoconjugated Pd (II) complex, [PdCl_2_ (L)], showed significant antitumor effects in CDDP-sensitive gastric cancer and executed their biological effects by inducing apoptosis. In addition, [PdCl_2_ (L)] overcame cross-resistance to CDDP in CDDP-resistant gastric cancer, while [PtCl_2_ (L)] did not. When compared with L-OHP, [PdCl_2_ (L)] showed a lower degree of cross-resistance to CDDP and [PdCl_2_ (L)] is speculated to be less toxic to the kidney than Pt complexes such as L-OHP and CDDP. Furthermore, glucose conjugation may increase drug solubility and tumor selectivity. From these findings, we conclude that [PdCl_2_ (L)] is a potentially useful antitumor drug for CDDP-resistant gastric cancer.

## Competing interests

All the authors declare that there is no conflict of interest.

## Authors’ contributions

Conception and design, MT and HK; Acquisition of data, MT; Analysis and interpretation of data, MT and HK; Drafting of the manuscript, MT and HK; Revising it critically for important intellectual content, SY, HO and KK; Final approval of the version to be published, HK and TJ; General supervision of research group, TJ. All authors read and approved the final manuscript.

## Pre-publication history

The pre-publication history for this paper can be accessed here:

http://www.biomedcentral.com/1471-2407/13/237/prepub

## References

[B1] Hainsworth JD Johnson DH Greco FA Cisplatin-based combination chemotherapy in the treatment of poorly differentiated carcinoma and poorly differentiated adenocarcinoma of unknown primary site: results of a 12-year experience J Clin Oncol 1992 10 6 912 922 1375284 10.1200/JCO.1992.10.6.912

[B2] Koizumi W Narahara H Hara T Takagane A Akiya T Takagi M Miyashita K Nishizaki T Kobayashi O Takiyama W Toh Y Nagaie T Takagi S Yamamura Y Yanaoka K Orita H Takeuchi M S-1 plus cisplatin versus S-1 alone for first-line treatment of advanced gastric cancer (SPIRITS trial): a phase III trial Lancet Oncol 2008 9 3 215 221 10.1016/S1470-2045(08)70035-4 18282805

[B3] Rennicke A Voigt W Mueller T Fruehauf A Schmoll HJ Beyer C Dempke W Resistance mechanisms following cisplatin and oxaliplatin treatment of the human teratocarcinoma cell line 2102EP Anticancer Res 2005 25 2A 1147 1155 15868958

[B4] Timmer-Bosscha H Mulder NH De Vries EG Modulation of cis-diamminedichloroplatinum(II) resistance: a review Br J Cancer 1992 66 2 227 238 10.1038/bjc.1992.249 1503895 PMC1977827

[B5] Kartalou M Essigmann JM Mechanisms of resistance to cisplatin Mutat Res 2001 478 1–2 23 43 11406167 10.1016/s0027-5107(01)00141-5

[B6] Hall MD Okabe M Shen DW Liang XJ Gottesman MM The role of cellular accumulation in determining sensitivity to platinum-based chemotherapy Annu Rev Pharmacol Toxicol 2008 48 495 535 10.1146/annurev.pharmtox.48.080907.180426 17937596

[B7] Brabec V Kasparkova J Modifications of DNA by platinum complexes Relation to resistance of tumors to platinum antitumor drugs. Drug Resist Updat 2005 8 3 131 146 10.1016/j.drup.2005.04.006 15894512

[B8] Florea AM Busselberg D Anti-cancer drugs interfere with intracellular calcium signaling NeuroToxicology 2009 30 5 803 810 10.1016/j.neuro.2009.04.014 19465052

[B9] Torigoe T Izumi H Ishiguchi H Yoshida Y Tanabe M Yoshida T Igarashi T Niina I Wakasugi T Imaizumi T Momii Y Kuwano M Kohno K Cisplatin resistance and transcription factors Curr Med Chem Anticancer Agents 2005 5 1 15 27 10.2174/1568011053352587 15720258

[B10] Xie XK Yang DS Ye ZM Tao HM Enhancement effect of adenovirus-mediated antisense c-myc and caffeine on the cytotoxicity of cisplatin in osteosarcoma cell lines Chemotherapy 2009 55 6 433 440 10.1159/000265527 19996588

[B11] Wang Z Xu B Lin D Tan W Leaw S Hong X Hu X XRCC1 polymorphisms and severe toxicity in lung cancer patients treated with cisplatin-based chemotherapy in Chinese population Lung Cancer 2008 62 1 99 104 10.1016/j.lungcan.2008.02.019 18400332

[B12] Jordan P Carmo-Fonseca M Molecular mechanisms involved in cisplatin cytotoxicity Cell Mol Life Sci 2000 57 8–9 1229 1235 11028915 10.1007/PL00000762 PMC11147023

[B13] Warburg O On the origin of cancer cells Science 1956 123 3191 309 314 10.1126/science.123.3191.309 13298683

[B14] Hirohara S Obata M Alitomo H Sharyo K Ando T Tanihara M Yano S Synthesis, photophysical properties and sugar-dependent in vitro photocytotoxicity of pyrrolidine-fused chlorins bearing S-glycosides J Photochem Photobiol B 2009 97 1 22 33 10.1016/j.jphotobiol.2009.07.007 19679489

[B15] Tanaka M Kataoka H Mabuchi M Sakuma S Takahashi S Tujii R Akashi H Ohi H Yano S Morita A Joh T Anticancer effects of novel photodynamic therapy with glycoconjugated chlorin for gastric and colon cancer Anticancer Res 2011 31 3 763 769 21498693

[B16] Brudzinska I Mikata Y Obata M Ohtsuki C Yano S Synthesis, structural characterization, and antitumor activity of palladium(II) complexes containing a sugar unit Bioorg Med Chem Lett 2004 14 10 2533 2536 10.1016/j.bmcl.2004.02.095 15109645

[B17] Dallavalle F Gaccioli F Franchi-Gazzola R Lanfranchi M Marchio L Pellinghelli MA Tegoni M Synthesis, molecular structure, solution equilibrium, and antiproliferative activity of thioxotriazoline and thioxotriazole complexes of copper II and palladium II J Inorg Biochem 2002 92 2 95 104 10.1016/S0162-0134(02)00545-7 12459154

[B18] Divsalar A Bagheri MJ Saboury AA Mansoori-Torshizi H Amani M Investigation on the interaction of newly designed anticancer Pd(II) complexes with different aliphatic tails and human serum albumin J Phys Chem B 2009 113 42 14035 14042 10.1021/jp904822n 19778061

[B19] Foster ER Downs JA Histone H2A phosphorylation in DNA double-strand break repair FEBS J 2005 272 13 3231 3240 10.1111/j.1742-4658.2005.04741.x 15978030

[B20] Grim J D'Amico A Frizelle S Zhou J Kratzke RA Curiel DT Adenovirus-mediated delivery of p16 to p16-deficient human bladder cancer cells confers chemoresistance to cisplatin and paclitaxel Clin Cancer Res 1997 3 12 Pt 1 2415 2423 9815642

[B21] Li YT Chua MJ Kunnath AP Chowdhury EH Reversing multidrug resistance in breast cancer cells by silencing ABC transporter genes with nanoparticle-facilitated delivery of target siRNAs Int J Nanomedicine 2012 7 2473 2481 22701315 10.2147/IJN.S30500 PMC3373294

[B22] Cole SP Sparks KE Fraser K Loe DW Grant CE Wilson GM Deeley RG Pharmacological characterization of multidrug resistant MRP-transfected human tumor cells Cancer Res 1994 54 22 5902 5910 7954421

[B23] Theou N Gil S Devocelle A Julie C Lavergne-Slove A Beauchet A Callard P Farinotti R Le Cesne A Lemoine A Faivre-Bonhomme L Emile JF Multidrug resistance proteins in gastrointestinal stromal tumors: site-dependent expression and initial response to imatinib Clin Cancer Res 2005 11 21 7593 7598 10.1158/1078-0432.CCR-05-0710 16278376

[B24] Vicent MJ Duncan R Polymer conjugates: nanosized medicines for treating cancer Trends Biotechnol 2006 24 1 39 47 10.1016/j.tibtech.2005.11.006 16307811

[B25] Liu Z Ballinger JR Rauth AM Bendayan R Wu XY Delivery of an anticancer drug and a chemosensitizer to murine breast sarcoma by intratumoral injection of sulfopropyl dextran microspheres J Pharm Pharmacol 2003 55 8 1063 1073 10.1211/0022357021567 12956895

[B26] Lin R Shi Ng L Wang CH In vitro study of anticancer drug doxorubicin in PLGA-based microparticles Biomaterials 2005 26 21 4476 4485 10.1016/j.biomaterials.2004.11.014 15701377

[B27] Foger F Noonpakdee W Loretz B Joojuntr S Salvenmoser W Thaler M Bernkop-Schnurch A Inhibition of malarial topoisomerase II in Plasmodium falciparum by antisense nanoparticles Int J Pharm 2006 319 1–2 139 146 16713146 10.1016/j.ijpharm.2006.03.034

[B28] Ambruosi A Khalansky AS Yamamoto H Gelperina SE Begley DJ Kreuter J Biodistribution of polysorbate 80-coated doxorubicin-loaded [14C]-poly(butyl cyanoacrylate) nanoparticles after intravenous administration to glioblastoma-bearing rats J Drug Target 2006 14 2 97 105 10.1080/10611860600636135 16608736

[B29] Dong Y Feng SS Nanoparticles of poly(D, L-lactide)/methoxy poly(ethylene glycol)-poly(D, L-lactide) blends for controlled release of paclitaxel J Biomed Mater Res A 2006 78 1 12 19 16596586 10.1002/jbm.a.30684

[B30] Farokhzad OC Karp JM Langer R Nanoparticle-aptamer bioconjugates for cancer targeting Expert Opin Drug Deliv 2006 3 3 311 324 10.1517/17425247.3.3.311 16640493

[B31] Cheng X Kuhn L Chemotherapy drug delivery from calcium phosphate nanoparticles Int J Nanomedicine 2007 2 4 667 674 18203433 PMC2676798

[B32] Raynaud FI Boxall FE Goddard PM Valenti M Jones M Murrer BA Abrams M Kelland LR: **cis**-**Amminedichloro**(**2**-**methylpyridine**) **platinum**(**II**) (**AMD473**), **a novel sterically hindered platinum complex**: **in vivo activity**, **toxicology**, **and pharmacokinetics in mice** Clin Cancer Res 1997 3 11 2063 2074 9815598

[B33] Yoshida M Khokhar AR Siddik ZH Cytotoxicity and tolerance to DNA adducts of alicyclic mixed amine platinum(II) homologs in tumor models sensitive and resistant to cisplatin or tetraplatin Oncol Rep 1998 5 5 1281 1287 9683851 10.3892/or.5.5.1281

[B34] Paul AK Srivastava TS Chavan SJ Chitnis MP Desai S Rao KK Synthesis, characterization, cytotoxic, and DNA binding studies of some platinum (II) complexes of 1,2-diamine and alpha-diimine with 2-pyridinecarboxylate anion J Inorg Biochem 1996 61 3 179 196 10.1016/0162-0134(95)00055-0 9064362

[B35] Monti E Gariboldi M Maiocchi A Marengo E Cassino C Gabano E Osella D Cytotoxicity of cis-platinum(II) conjugate models The effect of chelating arms and leaving groups on cytotoxicity: a quantitative structure-activity relationship approach. J Med Chem 2005 48 3 857 866 10.1021/jm049508t 15689170

[B36] Bierbach U Qu Y Hambley TW Peroutka J Nguyen HL Doedee M Farrell N Synthesis, Structure, Biological Activity, and DNA Binding of Platinum(II) Complexes of the Type trans-[PtCl(2)(NH(3))L] (L = Planar Nitrogen Base) Effect of L and Cis/Trans Isomerism on Sequence Specificity and Unwinding Properties Observed in Globally Platinated DNA. Inorg Chem 1999 38 15 3535 3542 10.1021/ic981181x 11671101

[B37] Ali MS Khan SR Ojima H Guzman IY Whitmire KH Siddik ZH Khokhar AR Model platinum nucleobase and nucleoside complexes and antitumor activity: X-ray crystal structure of PtIV(trans-1R,2R-diaminocyclohexane)trans-(acetate)2(9-ethylguanine)Cl]NO 3.H2O J Inorg Biochem 2005 99 3 795 804 10.1016/j.jinorgbio.2004.12.015 15708801

[B38] Coluccia M Natile G Trans-platinum complexes in cancer therapy Anticancer Agents Med Chem 2007 7 1 111 123 10.2174/187152007779314080 17266508

[B39] Manzotti C Pratesi G Menta E Di Domenico R Cavalletti E Fiebig HH Kelland LR Farrell N Polizzi D Supino R Pezzoni G Zunino F BBR 3464: a novel triplatinum complex, exhibiting a preclinical profile of antitumor efficacy different from cisplatin Clin Cancer Res 2000 6 7 2626 2634 10914703

[B40] Komeda S Kalayda GV Lutz M Spek AL Yamanaka Y Sato T Chikuma M Reedijk J New isomeric azine-bridged dinuclear platinum(II) complexes circumvent cross-resistance to cisplatin J Med Chem 2003 46 7 1210 1219 10.1021/jm020004+ 12646031

[B41] Zhu J Zhao Y Zhu Y Wu Z Lin M He W Wang Y Chen G Dong L Zhang J Lu Y Guo Z DNA cross-linking patterns induced by an antitumor-active trinuclear platinum complex and comparison with its dinuclear analogue Chemistry 2009 15 21 5245 5253 10.1002/chem.200900217 19350599

[B42] Kelland LR Murrer BA Abel G Giandomenico CM Mistry P Harrap KR Ammine/amine platinum(IV) dicarboxylates: a novel class of platinum complex exhibiting selective cytotoxicity to intrinsically cisplatin-resistant human ovarian carcinoma cell lines Cancer Res 1992 52 4 822 828 1737343

[B43] Yoshida M Khokhar AR Siddik ZH Axial ligands and alicyclic ring size modulate the activity and biochemical pharmacology of ammine/cycloalkylamine-platinum(IV) complexes in tumor cells resistant to cis-diamminedichloroplatinum(II) or trans-1R,2R-1S,2S-diaminocyclohexanetetrachloroplatinum(IV) Cancer Res 1994 54 17 4691 4697 8062266

[B44] Kelland LR Barnard CF Mellish KJ Jones M Goddard PM Valenti M Bryant A Murrer BA Harrap KR A novel trans-platinum coordination complex possessing in vitro and in vivo antitumor activity Cancer Res 1994 54 21 5618 5622 7923207

[B45] Caires AC Recent advances involving palladium (II) complexes for the cancer therapy Anticancer Agents Med Chem 2007 7 5 484 491 10.2174/187152007781668661 17896909

[B46] Chaney SG Campbell SL Bassett E Wu Y Recognition and processing of cisplatin- and oxaliplatin-DNA adducts Crit Rev Oncol Hematol 2005 53 1 3 11 10.1016/j.critrevonc.2004.08.008 15607931

[B47] Kruszewski M Bouzyk E Oldak T Samochocka K Fuks L Lewandowski W Fokt I Priebe W Differential toxic effect of cis-platinum(II) and palladium(II) chlorides complexed with methyl 3,4-diamine-2,3,4,6-tetradeoxy-alpha-L-lyxo-hexopyranoside in mouse lymphoma cell lines differing in DSB and NER repair ability Teratog Carcinog Mutagen 2003 Suppl 1 1 11 12616592 10.1002/tcm.10046

[B48] Tanaka T Huang X Halicka HD Zhao H Traganos F Albino AP Dai W Darzynkiewicz Z Cytometry of ATM activation and histone H2AX phosphorylation to estimate extent of DNA damage induced by exogenous agents Cytometry A 2007 71 9 648 661 17622968 10.1002/cyto.a.20426 PMC3855668

[B49] Ewald B Sampath D Plunkett W H2AX phosphorylation marks gemcitabine-induced stalled replication forks and their collapse upon S-phase checkpoint abrogation Mol Cancer Ther 2007 6 4 1239 1248 10.1158/1535-7163.MCT-06-0633 17406032

[B50] Huang X Okafuji M Traganos F Luther E Holden E Darzynkiewicz Z Assessment of histone H2AX phosphorylation induced by DNA topoisomerase I and II inhibitors topotecan and mitoxantrone and by the DNA cross-linking agent cisplatin Cytometry A 2004 58 2 99 110 15057963 10.1002/cyto.a.20018

[B51] Banath JP Olive PL Expression of phosphorylated histone H2AX as a surrogate of cell killing by drugs that create DNA double-strand breaks Cancer Res 2003 63 15 4347 4350 12907603

[B52] Olive PL Banath JP Kinetics of H2AX phosphorylation after exposure to cisplatin Cytometry B Clin Cytom 2009 76 2 79 90 18727058 10.1002/cyto.b.20450

[B53] Niedernhofer LJ Odijk H Budzowska M Van Drunen E Maas A Theil AF De Wit J Jaspers NG Beverloo HB Hoeijmakers JH Kanaar R The structure-specific endonuclease Ercc1-Xpf is required to resolve DNA interstrand cross-link-induced double-strand breaks Mol Cell Biol 2004 24 13 5776 5787 10.1128/MCB.24.13.5776-5787.2004 15199134 PMC480908

[B54] Banuelos CA Banath JP Kim JY Aquino-Parsons C Olive PL gammaH2AX expression in tumors exposed to cisplatin and fractionated irradiation Clin Cancer Res 2009 15 10 3344 3353 10.1158/1078-0432.CCR-08-3114 19401347

